# Diagnosis of periprosthesis joint infection and selection of replantation timing: a novel nomogram diagnosis model

**DOI:** 10.1097/JS9.0000000000002574

**Published:** 2025-06-12

**Authors:** Wenpeng Jin, Yunbo Du, Xianqiang Liu

**Affiliations:** a Intensive Care Unit, Shenzhen Longhua District Central Hospital, Shenzhen, Guangdong, China; b Medical School of Chinese PLA, Beijing, China

We read with great interest the recent article published in your journal regarding a nomogram-based diagnostic model for periprosthetic joint infection (PJI)^[1]^. The study integrated multiple serological and synovial biomarkers to construct a clinically applicable prediction tool, aiming to support preoperative identification of PJI. The authors should be commended for their efforts in addressing this complex clinical issue.

However, after a thorough analysis of the manuscript, we believe several critical aspects merit further discussion. Our comments are as follows:The authors reported an AUC of 0.998 in the training cohort and 0.997 in the validation cohort. Such near-perfect classification performance is exceptionally rare in real-world clinical scenarios. Given that the model was developed using a retrospective dataset with numerous variables and potential interactions, the remarkably high accuracy without external, large-scale validation may suggest overfitting, rather than genuine generalizability.The study employed LASSO regression to select 13 potential predictors, ultimately including 8 variables in the nomogram (e.g., SE-IL-6, CD64, SF-CRP). We noticed that some of these variables, such as CD64 index and synovial IL-4, are not commonly recommended in current PJI diagnostic guidelines. The manuscript lacks a comprehensive discussion of the clinical rationale and biological mechanisms underlying these novel biomarkers. Furthermore, there is no report of multicollinearity testing among predictors, which weakens the interpretability and credibility of the model.While the authors claim their model outperforms the MSIS (2013) and ICM (2018) criteria, they did not provide complete comparative data, including sensitivity, specificity, positive/negative predictive values, or subgroup analysis (e.g., acute vs. chronic infection, hip vs. knee joints). Without a comprehensive comparison under specific clinical scenarios, it is difficult for readers to fully assess whether the new model can truly replace or complement existing standards in routine practice.

In conclusion, this study represents a valuable step forward in exploring novel approaches to PJI diagnosis. However, we suggest the authors consider expanding their data sources, reinforcing multicenter prospective validation, and providing more robust explanations of variable function and clinical significance to enhance the model’s clinical utility. Addressing these issues would greatly strengthen the study’s impact and relevance for broader clinical application. We appreciate the authors’ contribution to this important field and look forward to continued dialogue that may further improve the clinical translation of this model.

## Data Availability

Not applicable.
